# The lure of novel biological and chemical entities in food-system transformations

**DOI:** 10.1016/j.oneear.2022.09.011

**Published:** 2022-10-21

**Authors:** Peter Søgaard Jørgensen, Daniel I. Avila Ortega, Robert Blasiak, Sarah Cornell, Line J. Gordon, Magnus Nyström, Per Olsson

**Affiliations:** 1 Stockholm Resilience Centre, Stockholm University, Stockholm, Sweden; 2 Global Economic Dynamics and the Biosphere Programme, Royal Swedish Academy of Sciences, Stockholm, Sweden; 3 Graduate School of Agricultural and Life Sciences, The University of Tokyo, Bunkyo-ku, Tokyo, Japan

## Abstract

Synthetic chemicals and biologically engineered materials are major forces in today’s food systems, but they are also major drivers of the global environmental changes and health challenges that characterize the Anthropocene. To address these challenges, we will need to increase assessment activity, promote alternative production practices with less reliance on such technologies, and regulate social campaigns and experiments.

## Main text

Novel entities (NEs) such as synthetic chemicals, engineered materials, and genetically modified organisms have been key drivers of exponential socio-economic growth since at least the so-called Green Revolution of the 1950s, which drove the spread of technology-driven agriculture around the world. But NEs and the practices they have supported are also associated with major negative impacts on the global environment and human health. For example, synthetic fertilizers, pesticides, and plastics that are essential in conventional production strategies and a cornerstone of today’s global food security paradigm are also major contributors to climate change, biodiversity loss, eutrophication, and pollution of environments with xenobiotics.

The innovative and rapidly changing nature of NEs means that defining a planetary boundary for them has challenged scientists for more than a decade. However, regardless of whether one opts for a definition at an Earth-systems scale or a more human-oriented definition, scientists agree that we have moved beyond an operating space that is safe for NEs. The major forces behind this conclusion are the rapid growth in diversity of types and volumes of NEs in the environment combined with a lack of assessment activity and capacity.^1^^,^^2^

**Figure 1:**
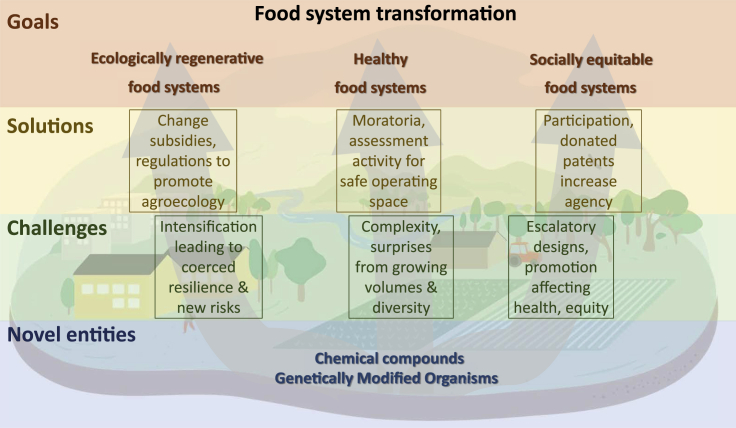
A transformative pathway toward sustainable food systems that address some of the major challenges of novel entities

Building ecologically regenerative, socially empowered, and healthy food systems are central goals of sustainability transformations. Although, NEs to a large extent facilitate adaptation to changing conditions, the lure of incremental adaptation of current food system practices can ultimately hinder or delay the necessary transformative shift toward a sustainability agenda. Does this mean that technological developments, such as our capacity to build synthetic biological systems, perform precision gene editing, unleash gene drive cascades to suppress pests and pathogens, and similar technologies to genetically modify living organisms will play no role in this new transformative agenda (Box 1)? This is the conundrum we are faced with: our capacity to wield novel entities, both tested and untested at scale, has never been greater, but their role in moving toward the goal of creating sustainable and healthy food systems has never been more uncertain. To realign NEs with food-system transformations, a layer of challenges will have to be addressed (Figure 1).

Box 1 **Genetically modified organisms as novel entities**

In the context of the global food system, humanity benefits from thousands of years of selective breeding and crossbreeding of plants, livestock, and microorganisms promoting desired traits. Since the 1970s, it has become possible to use biotechnology to directly alter DNA to create genetically modified organisms (GMOs), and by 1994, the first GMO produce (tomatoes) were approved for sale in the United States. Today, over 10% of the Earth’s arable land is planted with GMOs, including over 94% of soybeans and 92% of corn. Cows have been genetically modified for disease resistance, lack of horns, and other traits, while transgenic Atlantic salmon (*Salmo salar*) includes genes from Chinook salmon (*Oncorhynchus tshawytscha*) and the ocean pout (*Zoarces americanus*), enabling it to reach market size in half the time, and to persist in near-freezing conditions. In several parts of the world, large-scale salmon aquaculture operations overlap with wild salmon populations, and escapement of farmed salmon into these environments threatens the genetic identity of wild populations.

The advent of the CRISPR technique in 2012 dramatically reduced the cost and complexity of gene editing (do-it-yourself CRISPR kits now cost less than USD 500) and comes alongside rapid advances in gene sequencing technologies and associated bioinformatics tools. This has resulted, for instance, in the average cost of sequencing a base pair of DNA falling by five orders of magnitude in twenty years, from USD 6,000 in 2001 to USD 0.01 today. Databases of genetic sequence data like GenBank’s Sequence Read Archive have been growing exponentially, doubling in size roughly every 18 months since the 1980s, providing a vast library of publicly accessible genetic data that forms the basis for modern biotechnology. The entry of genetic sequence data from the Earth’s biodiversity into these public databases raises questions about equity and protection of national and cultural heritage, as no obligations currently exist (e.g., for companies) to share benefits from use of genetic sequence data, and access to much of this data (e.g., in GenBank) is currently unrestricted. The Nagoya Protocol (2014), which was established in part to eliminate egregious forms of biopiracy, deals with physical samples, and negotiations are underway under the aegis of the Convention on Biological Diversity to consider the extent to which digital sequence information (another term for genetic sequence data) can be subject to corresponding access and benefit sharing obligations.

### The lure of novel entities

In the short term, NEs have become essential to maintain high and predictable yields within conventionally intensified food systems. This is facilitated by a stream of new and often cheaper source of fertilizers, biocontrol, or disease-resistance mechanisms. With time, however, the reliance on NEs often grows as initial stocks of ecosystem services are depleted, such as soil organic content and soil microbiomes, or new biocontrol mechanisms are needed as pests evolve resistance to pesticides and antibiotics. Similarly, because NEs are often used as a means to increase yield while reducing labor costs, they risk eroding social resilience, i.e., the ability to cooperate at appropriate scales in the face of uncertainty, as fewer people are involved in the operation. As such, NEs contribute to what has been coined “coerced resilience” in production ecosystems.^3^

Although the above is a caricature of much more complex dynamics in the real world, this coerced resilience is a significant phenomenon underlying the current state of play in the global production ecosystem.^3^ The present energy and food security crisis clearly illustrates the risks of locking into an NE intensive food production. That is because production of most NEs require significant amounts of energy. Thus, when energy prices increase, so do fertilizer and pesticide prices. Take ammonia for example: BASF, Europe’s largest producer of ammonia for nitrogen fertilizer, plastics, and other products has had to cut production of these NEs as Russia has lowered its supply of gas.^4^ The current supply and energy crisis is therefore a real test of food-system resilience around and will likely provide insights into the trade-offs between systems with high and low dependence on NEs.

Emerging pests and pathogens (EPPs) are another example of the consequences of intensified systems propped up by NEs. Although the COVID-19 pandemic helped draw attention to the consequences of how we manage global land area, the challenge is in fact much broader than human pathogens and also includes weeds, invertebrates, and microbial pathogens in food systems.^4^^,^^5^ In fact, intensified systems have the capacity to generate entirely new epidemics by changing the nature of interactions between domesticated species and wild organisms from beneficial to pathogenic. They do so by making virulent life cycles less costly to wild organisms,^4^ and as it only takes a small change in their genetic composition to go from friend to foe, such transitions are of increasing concern. For example, the horizontal transfer of a single plasmid is enough to turn beneficial plant mutualists into pathogens and largely commensal bacteria into hard-to-treat, virulent human infections.^5^ The subsequent spread of these endogenous EPPs is facilitated by large homogeneous landscapes or via trade networks, and once widely spread, EPPs can enter a diversification cycle of repeated evolution, not unlike SARS-CoV2.^1^

#### The ripple effect

As the use and effects of NEs tend to accumulate over time, the challenge of governing NEs keeps increasing in complexity as new NEs are introduced. There is concern not only over the NEs used in food systems but also those used outside the immediate food system. The latter category includes pollutants affecting soil, water, and wildlife that in some way or form are part of food systems, whether as consumed goods or essential stock of ecosystem services. We can think of each NE as a drop of water creating rings of cascading change in clear calm lake and the paths and interactions of rings become increasingly hard to predict as the number of drops increases. Food systems in the Anthropocene are best described as a turbulent lake in the midst of a persistent cloudburst. The challenge of predicting the long-term effects of these myriad interactions is as real for individual human health as it is for Earth system scale dynamics.

Take the example of the food additive disaccharide Trehalose commonly used in prepared frozen foods, like ice cream, and the bacterium *Clostridioides difficile*, a common cause of human gut infection following antibiotic treatment. Trehalose was recently argued to have played a role in creating more infectious and difficult-to-treat variants of *C. difficile*.^6^ Following the approval of Trehalose by the US Food and Drug Administration, two strains of *C. difficile* acquired unique mechanisms to metabolize low concentrations of the disaccharide trehalose, which increased their sensitivity to the compound more than 500-fold and were experimentally shown to increase their virulence in the presence of Trehalose in mice. Globally these two strains are now spreading and are accounting for an increasing proportion of the more than 200,000 thousand cases and more than 12,000 deaths caused by the bacterium in hospitals in the USA alone every year. It is probable that this NE had highly surprising effects on one important pathogen of concern. However, many—and perhaps most—of such cascading effects of NEs may never be discovered, due to difficulties in tracing them and proving causality.

#### The social dimension of novel entities

The uncertainty concerning NEs in food-system transformations goes far beyond the bewildering number and increasing volumes of such compounds. NEs can be implemented in multiple product designs and promoted with widely different social marketing strategies. Although the screening challenge is immense and the call for moratoriums of both chemical^7^ and biological^8^^,^^9^ NEs are growing, there are certain types of NE product and marketing designs that require special attention. A poster child illustrating this point is the aggressive promotion of the world’s most used herbicide, glyphosate. A concentrated market and vigorous promotion across multiple sectors characterized the marketing of the herbicide and likely increased its global use in gardening among the general public as well as among professional farmers.^10^ One example is the widespread adoption of glyphosate-resistant transgenic crops that allow farmers to spray weeds more indiscriminately without affecting their crop. The rapid and indiscriminate increase in use means that resistance to glyphosate is now found in 56 crops across all continents with agricultural production. The negative health impacts of this otherwise comparatively low toxicity herbicide (dosage-wise) have recently been the focus in the context of non-Hodgkin’s lymphoma cancers. A class-action lawsuit involving more than 100,000 cases was filed and has resulted in large settlements with more than USD 11 billion paid in settlements to date by the former producer of the NE. In the meantime, new transgenic crops with combined resistance to glyphosate and the major alternative herbicides dicamba and 2,4-D are increasingly adopted as the efficacy of glyphosate declines. Glyphosate’s approval in the EU is up for evaluation in December 2022. As with antibiotic resistance, the key problem here is that companies’ earnings are linked to sales of a transgenic crop and pesticide that depletes an exhaustible biological resource: the susceptibility of living organisms.^1^

### A transformative pathway

The first priority for aligning NEs with goals of ecologically regenerative and healthy food systems and the re-building of social resilience in food systems is to move back within the safe operating space of novel entities (Figure 1). Doing so hinges critically on gaining a better understanding of the effects of novel entities on human health, wellbeing, and the environment, including the Earth system. In order to reduce these planetary pressures, the introduction of additional NEs can be prevented until long-term impacts of current technologies are better assessed. Recently, a group of authors argued that our scientific understanding of such effects for chemical NEs is so limited that the world should seriously consider a moratorium on additional NEs until we have a better assessment of the combined effects of those that are currently in use.^7^ The need for governance transformations that supports new approaches to screening and evaluation mechanisms for long-term unintended consequences is clear and the recent suggestion of an international science-policy body on chemicals and waste could, especially if expanded to include other NEs, be a first step in that direction at a near global scale.^2^

Alternative production strategies in food systems will be needed while we increase our knowledge of the effects of NEs on people and planet that help build ecological and social resilience in production systems while maintaining yields. There is therefore great encouragement to be found in recent studies showing that ecological intensified (e.g., organic or agro-ecological production forms) can compete with conventional production practices in the long term, and while doing so, deliver additional ecosystem services, such as pollination, maintenance of soil fertility, and biological control, as well as offer employment opportunities in the form of increased labor.^11^ These findings, in principle, refute the old food security paradigm of conventional intensification as well as the concern that ecological intensification will lead to continued biodiversity loss through expanded land and ocean use. However, to encourage increased uptake of such practices, subsidies, regulations, and standards will need to be shifted away from environmentally harmful practices and technologies toward practices such as inter-cropping, agro-ecological farming and integrated pest management.^12^

### Co-owned and participatory designs

It would, however, be unwise to shut the door completely on NEs. Instead, we need to identify the opportunities for NEs to provide short-term benefits without compromising long-term sustainability. For example, supplying Vitamin A or its precursor beta-carotene to highly undernourished settings has been seen as a quick way to move toward achieving goals of nutritional health in children and avoiding illnesses such as blindness. Although transgenic crops again have been promoted as an alternative solution, partly due to the long 20-year wait for the approval of Golden Rice (a variety of rice produced through genetic engineering to biosynthesize beta-carotene, a precursor of vitamin A), there remains a lack of evidence for the extent to which transgenic crops could prove superior to other ways of supplying Vitamin A, with one risk being side effects from overconsumption.^13^ The uncertainty associated with whether such crops can be locally bred and involved in informal seed-exchange and breeding programs, a key source of social resilience in some regions, is another concern. However, in the case of Golden Rice, this problem seems to have been in part overcome by donating the patents to the Golden Rice Humanitarian Board, which allows farmers to further breed and interbreed the variety to adapt it to local settings. A recent trend to further enable participatory and co-owned approaches of small-scale producers and consumers is the ambition to co-produce strategies for the implementation of biological NEs, such as synthetic gene-drive organisms, e.g., for malaria control. However, such ambitions can be vulnerable to slippage on commitments, meaning that governance systems that support extreme care, stringent follow up and exit clauses should be considered as routine parts of such experiments.^14^ Nonetheless, such steps to ensure social-ecological resilience of food systems in the context of NEs provide some hope for their future use.

#### De-linkage of sales from earnings

Clearly other interventions are needed that can outpace the slowness of settling regulatory issues of transgenic crops and avoid the risk of failing on still loose promises of participatory implementation of NEs in food systems. One such intervention is de-linkage of company earnings from sales, to avoid overconsumption as well as lack of innovation of NEs that are tied to exhaustible biological resources, such as pest and pathogen susceptibility. The failure to de-link sales and earnings has been a persistent driver behind the antibiotic resistance crisis as many companies moved to more profitable markets of other medical products. In the meantime, societies were still locked into a model of high antibiotic reliability thus continuing to erode the finite stock of antibiotic susceptibility among commensal as well as pathogenic bacteria.^1^ In the run up to the UN General Assembly’s High-Level meeting on Antimicrobial Resistance in 2016, de-linkage was proposed as one central solution to both the innovation and overconsumption problem and de-linkage will be key to address the much less-well-monitored problems of pesticide reliance.

### Conclusion

Whether society in the future chooses a more restrictive or a more optimistic approach to the role of NEs in food system transformations, they must be considered much more explicitly in future goal setting strategies, such as a Sustainable Development Goals (SDGs) 2.0 process, compared to the fragmented consideration of chemicals in the current SDGs agenda.^15^ One place to do so is in the new 2030 framework for biodiversity and associated processes under the umbrella of the Convention on Biological Diversity aimed at ensuring equitable access and sharing of benefits from genetic resources, including digital sequence information, which is expected to grow increasingly central in biotechnology activities. In order to navigate transformations toward food systems that enhance sustainability, equity, and human health, work should begin now to build scientific, political, and public capacity for the explicit consideration of NEs in designing sustainable development pathways.
